# OGG1 Inhibition Reduces Acinar Cell Injury in a Mouse Model of Acute Pancreatitis

**DOI:** 10.3390/biomedicines10102543

**Published:** 2022-10-12

**Authors:** Zoltán Hajnády, Máté Nagy-Pénzes, Máté A. Demény, Katalin Kovács, Tarek El-Hamoly, József Maléth, Péter Hegyi, Zsuzsanna Polgár, Csaba Hegedűs, László Virág

**Affiliations:** 1 Department of Medical Chemistry, Faculty of Medicine, University of Debrecen, 4032 Debrecen, Hungary; 2 Doctoral School of Molecular Medicine, University of Debrecen, 4032 Debrecen, Hungary; 3 MTA-DE Cell Biology and Signaling Research Group, 4032 Debrecen, Hungary; 4 Drug Radiation Research Department, National Centre for Radiation Research and Technology, Atomic Energy Authority, Cairo 11787, Egypt; 5 First Department of Medicine, University of Szeged, 6720 Szeged, Hungary; 6 HAS-USZ Momentum Epithelial Cell Signaling and Secretion Research Group, 6720 Szeged, Hungary; 7 Department of Public Health, University of Szeged, 6720 Szeged, Hungary; 8 Institute for Translational Medicine, János Szentágothai Research Centre, University of Pécs Medical School, 7624 Pécs, Hungary; 9 Momentum Gastroenterology Multidisciplinary Research Group, Hungarian Academy of Sciences, University of Szeged, 6720 Szeged, Hungary

**Keywords:** acute pancreatitis, 8-oxoguanine glycosylase, inflammation, cell death, necrosis, oxidative stress, poly(ADP-ribose) polymerase 1

## Abstract

Acute pancreatitis (AP) is a potentially life-threatening gastrointestinal disease with a complex pathology including oxidative stress. Oxidative stress triggers oxidative DNA lesions such as formation of 7,8-dihydro-8-oxo-2′-oxoguanine (8-oxoG) and also causes DNA strand breaks. DNA breaks can activate the nuclear enzyme poly(ADP-ribose) polymerase 1 (PARP1) which contributes to AP pathology. 8-oxoG is recognized by 8-oxoG glycosylase 1 (OGG1) resulting in the removal of 8-oxoG from DNA as an initial step of base excision repair. Since OGG1 also possesses a DNA nicking activity, OGG1 activation may also trigger PARP1 activation. In the present study we investigated the role played by OGG1 in AP. We found that the OGG1 inhibitor compound TH5487 reduced edema formation, inflammatory cell migration and necrosis in a cerulein-induced AP model in mice. Moreover, TH5487 caused 8-oxoG accumulation and reduced tissue poly(ADP-ribose) levels. Consistent with the indirect PARP inhibitory effect, TH5487 shifted necrotic cell death (LDH release and Sytox green uptake) towards apoptosis (caspase activity) in isolated pancreatic acinar cells. In the in vivo AP model, TH5487 treatment suppressed the expression of various cytokine and chemokine mRNAs such as those of TNF, IL-1β, IL1ra, IL6, IL16, IL23, CSF, CCL2, CCL4, CCL12, IL10 and TREM as measured with a cytokine array and verified by RT-qPCR. As a potential mechanism underlying the transcriptional inhibitory effect of the OGG1 inhibitor we showed that while 8-oxoG accumulation in the DNA facilitates NF-κB binding to its consensus sequence, when OGG1 is inhibited, target site occupancy of NF-κB is impaired. In summary, OGG1 inhibition provides protection from tissue injury in AP and these effects are likely due to interference with the PARP1 and NF-κB activation pathways.

## 1. Introduction

Acute pancreatitis (AP) is an acute inflammatory disease of the pancreas. With an incidence of 34/100,000 cases per year in wealthy countries [1], AP is one of the most common gastrointestinal causes of hospital admissions. The incidence of AP is higher in the American region compared to the European and Western Pacific region [1] and the treatment costs of AP place a significant burden on health insurance systems worldwide. The most common causes of AP are gall stones, alcohol consumption, hypertriglyceridemia, biliary obstruction and endoscopic retrograde cholangiopancreatography. However, many other etiologic factors (e.g., medications, pancreatic or Vater papilla tumor, trauma, hypercalcemia, various infections) are also known. In 10–20% of cases, however, no underlying cause can be identified [2]. The severity of AP ranges from mild to fulminant, and the mortality of the disease is high. Currently, there is no specific therapy for AP and therefore the search for new therapeutic targets is of utmost importance to reduce mortality.

Although several cellular and molecular events appear to be important for the development of AP, the pathomechanism of the disease is not fully understood. The known critical events include early activation of trypsinogen, disturbances of ductal bicarbonate and fluid secretion, activation of nuclear factor kappa-B (NF-κB), early acinar mitochondrial damage and oxidative stress [3,4,5,6,7]. Overproduction of reactive oxygen or nitrogen species (ROS and RNS, respectively), insufficient antioxidant defense and consequential oxidative tissue injury have often been observed in AP [8]. Oxidative stress causes protein oxidation, lipid peroxidation and it also targets DNA causing single strand breaks and guanine oxidation (7,8-dihydro-8-oxo-2′-oxoguanine (8-oxoG) formation). Guanine can be oxidized by hydroxyl or alkylperoxyl radicals and singlet oxygen both in DNA and in dGTP [9]. DNA breaks can activate the nuclear enzyme poly(ADP-ribose) polymerase 1 (PARP1) which may contribute to AP pathology [10,11,12]. Guanine oxidation is recognized by 8-oxoguanine glycosylase 1 (OGG1) resulting in the removal of 8-oxoG from DNA as an initial step of base excision repair followed by DNA strand incision by apurinic/apyrimidinic endonuclease-1 (APE1) [13]. Since OGG1 also possesses a DNA nicking activity, OGG1 activation may trigger PARP1 activation [14]. In lymphocytes and HeLa cells, approximately one in a million guanines is estimated to be in the oxidized form (background level of base oxidation) [15] and efficient repair of 8-oxoG is of utmost importance as this lesion is considered mutagenic and has been linked to carcinogenesis and to degenerative diseases [16,17].

OGG1 has also been linked to the inflammatory response. On the one hand OGG1 deficient mice have been shown to develop less severe inflammation in contact hypersensitivity (dermatitis) and endotoxin shock [18]. Furthermore, siRNA-mediated depletion of OGG1 from airway epithelium suppressed allergic lung inflammation [19]. NF-κB activation and MAP kinase signaling have been identified as key molecular mechanisms underlying the proinflammatory role of OGG1 [20]. Recently, a small molecule OGG1 inhibitor compound was also developed and was found to dampen inflammation and decrease DNA occupancy of the master regulator of inflammation NF-κB. However, the role of OGG1 in AP has not yet been studied.

Here we set out to investigate whether OGG1 contributes to inflammation in AP. Using the OGG1 inhibitor TH5487, we found that the drug suppressed acinar injury and inflammation in a mouse model of cerulein-induced AP. Critical events in the anti-inflammatory effect of OGG1 inhibition involve prevention of PARP1 activation. inhibition of PARP1-mediated necrotic cell death and NF-κB activation.

## 2. Materials and Methods

### 2.1. Reagents

Cerulein was purchased from Medchemexpress LLC (Monmouth Junction, NJ, USA). TH5487 was purchased from Axon Medchem LLC (Reston, VA, USA). Alpha-amylase and lipase enzymatic assay kits were purchased from Diagnosticum Zrt (Budapest, Hungary). Dulbecco’s Modified Eagle Medium (DMEM), potassium bromate (KBrO3), ribonuclease A (RNAse), hexadecyltrimethylammonium bromide (HTAB), 3,3′,5,5′-tetramethylbenzidine (TMB), protease inhibitor cocktail and hydrogen peroxide, calcein-AM were purchased from Sigma–Aldrich (St. Louis, MI, USA). Penicillin–streptomycin was from Lonza (Basel, Switzerland). NucleoSpin RNA Plus kit was purchased from Macherey-Nagel (Düren, Germany). Proteome Profiler Mouse Cytokine Array kit and CometSlide High-Throughput kit were purchased from R&D Systems (Minneapolis, MN, USA). High-capacity cDNA reverse transcription kit was from Applied Biosystems (Foster City, CA, USA) and 2X qPCRBIO Sygreen Mix Lo-ROX was obtained from PCR Biosystems (London, UK). Anti-8-hydroxy-2′-deoxyguanosine antibody (ab48508) and cleaved-PARP1 antibody (ab32064) were purchased from Abcam (Cambridge, UK)). LightShift chemiluminescent EMSA kit, CellEvent Caspase-3/7 green reagent, DRAQ5 and Sytox Green nucleic acid stains were purchased from Thermo Fisher Scientific (Waltham, MA, USA). One step polymer HRP were from Biogenex (Fremont, CA, USA). Nuclear Fast Red Counterstain and Mouse-on-Mouse (MOM) kit were from Vector Laboratories (Burlingame, CA, USA). The anti-PAR antibody was purified from the culture medium of the 10 H hybridoma clone. Anti-NF-κB p65 antibody (8242) was from Cell Signaling (Danvers, MA, USA) and LDH assay was purchased from G-Biosciences (St. Louis, MO, USA).

### 2.2. Acute Pancreatitis Model

Animal experiments were approved by the institutional animal welfare committee (25/2017/DEMÁB). Mice (C57BL/6j, 8–10 weeks old) were bred and maintained at 21–23 °C, 30–60% relative humidity, and a 12 h light/dark cycle. Mice housed in Eurostandard Type II cages were fed ad-libitum with VRF1 (P) food and water. Acute pancreatitis was induced as previously described [21] with modification as detailed in [22]. Briefly male mice (*n* = 18) were randomized into three groups: control, cerulein, and cerulein + TH5487. AP was induced by eight intraperitoneal injections of cerulein (50 μg/kg BW) at hourly intervals while mice in the control group were given saline only. TH5487 was administered in a single i.p. injection of 30 mg/kg BW 1 h before the first cerulein injection. TH5487 was dissolved in dimethyl sulfoxide (DMSO) and diluted in saline containing 10% Tween 80 as previously described [23]. Saline containing 5% DMSO, 10% Tween 80 was used as vehicle for control and cerulein groups. In a separate line of experiments, we confirmed that in these concentrations neither DMSO nor Tween20 had any effect on the measured parameters (data not shown). Mice were sacrificed 10 h after the first cerulein injection and blood and pancreas tissues were collected. Pancreata were either fixed in 4% formaldehyde or snap-frozen in liquid nitrogen and stored at −70 °C until analyzed.

### 2.3. Isolation and Culture of Pancreatic Acinar Cells

Pancreatic acinar cells were isolated from male C57BL/6j mice and cultured in high glucose Dulbecco’s modified Eagle’s medium supplemented with 5% fetal bovine serum, 1% penicillin-streptomycin mixture, 0.25 mg/mL of trypsin inhibitor (Sigma-Aldrich, St. Louis, MO, USA), and 25 ng/mL of recombinant human epidermal growth factor (Sigma-Aldrich, St. Louis, MO, USA) as described previously [22].

### 2.4. Serum α-Amylase and Lipase Determination

Blood samples were taken by cardiac puncture and were centrifuged at 5000× *g* for 10 min at 4 °C. Serum α-amylase and lipase activities were measured using enzymatic assay kits as previously described [22].

### 2.5. Myeloperoxidase Assay

Tissue myeloperoxidase (MPO) activity was measured as an indicator of neutrophil infiltration in the pancreas, as described previously [24] with modifications as detailed in [22].

### 2.6. Histology

Pancreatic tissue was collected and fixed in 10% formalin for 48 h. Dehydration, embedding, sectioning, and staining with hematoxylin and eosin (H&E) were performed according to guidelines in [25]. Staining was scored by a pathologist blinded to the experimental design and identity of the samples. A scoring scheme from 0 to 3 for edema, leukocyte infiltration, and necrosis was used, as previously described [22].

### 2.7. Immunohistochemistry

For 8-oxoG staining, antigen retrieval was performed in 10 mM citrate buffer (pH 6.0). Slides were then soaked in 4 N HCl and pH was neutralized in 50 mM Tris-base for 5 min at room temperature. For cleaved PARP staining antigen retrieval was performed in Tris/EDTA buffer pH 9.0. All sections were blocked with 5% FBS in PBS containing 0.2% Triton X-100 for 30 min Slides were then incubated with primary antibody against 8-oxodG (1:150) or cleaved PARP1 (1:100) at 4 °C overnight. Slides were washed three times with PBS and were then incubated with the peroxidase-conjugated secondary antibody according to the manufacturer’s instructions for 1 h. Diaminobenzidine (DAB) substrate was added to the slides for 5 min and 0.5% CoCl_2_ was added to enhance the signal. Nuclear fast red was used to counterstain cells. The DAB signal was analyzed with the QuPath software.

### 2.8. Immunofluorescent Staining

8-oxoG staining was performed as described previously [26] with modifications as follows. AR42J rat pancreatic acinar cells were cultured at 37 °C in a 5% CO2 atmosphere in DMEM supplemented with 20% of fetal bovine serum and 1% penicillin/streptomycin. To induce oxidative DNA damage, cells were treated with 30 mM potassium bromate (prepared in serum-free medium) for 1 h. Cells were then allowed to recover in medium containing either 0.1% DMSO or 10 µM TH5487 for 2 h. Cells were fixed with the mixture of ice-cold acetone:methanol (1:1) for 20 min at −4 °C, hydrated in PBS and permeabilized with 0.5% Triton X-100 in PBS. This was followed by 100 µg/mL RNAse treatment (for 1 h at 37 °C) to degrade RNAs. DNA was denatured by incubating cells in 2.5 N HCl for 20 min at RT, neutralized with 50 mM Tris-base and blocked with 2% bovine serum albumin in PBS for 1 h before proceeding to primary antibody incubation against 8-oxodG (1:100; for 2 h at RT). Incubation with the secondary antibody Alexa 633 (1:500) was performed for 1 h at RT. DAPI was used to stain DNA. Immunofluorescent 8-oxoG staining of tissue section was performed by M.O.M. Immunodetection kit according to the manufacturer’s instructions. Immunofluorescent PAR staining was performed as previously described [22]. Cells and sections were viewed with a Leica Sp8 Confocal Microscope (Leica, Wetzlar, Germany) and fluorescence intensities were analyzed using Cell Profiler software.

### 2.9. Cytokine Array

Cytokine and chemokine contents of the pancreata were measured using Proteome Profiler Mouse Cytokine Array Kits. Pancreatic lysates from 3 animals/treatment group were pooled together and prepared as described in the manufacturer’s instructions. Briefly, pancreatic tissues were harvested and homogenized in lysis solution supplemented with protease inhibitor cocktail and centrifuged at 10,000× *g* for 15 min at 4 °C. The supernatant was collected and applied to Mouse Cytokine Array Panel membrane. The membranes were visualized using ChemiDoc™ Imaging System by Bio-Rad. The pixel intensity was quantitated with the ImageLab software (Bio-Rad).

### 2.10. RNA Isolation and Quantitative Real-Time PCR

Isolation of total RNA as well as reverse transcription and quantitative real-time PCR conditions were the same as previously described [22,27]. Gene expression levels were normalized to the harmonic mean of RPS26 and RPLP0 as reference housekeeping genes. The primer sequences used for RT-qPCR are summarized in Table 1.

### 2.11. Electrophoretic Mobility Shift Assay (EMSA)

EMSA assays were performed using LightShift Chemiluminescent EMSA kits (Thermo-Fisher Scientific, Waltham, MA, USA) according to the manufacturer’s instructions. Briefly, nuclear extracts prepared from pancreata were incubated with biotin labelled double stranded DNA derived from the CCL2 promoter region that contains the NF-κB binding element. The probe was modified with 8-oxoG (sense: 5-CCCG*AAGGGTCTGGGAACTTCCAATACTGCC-3 antisense: 5-GGCAGTATTGGAAGTTCCCAGACCCTTCGGG-3, G* signifies the inclusion of 8-oxoG whereas the underlined sequence indicates the NF-κB binding region) or was synthesized without modifications (sense: 5-CCCGAAGGGTCTGGGAACTTCCAATACTGCC-3, antisense: 5-GGCAGTATTGGAAGTTCCC AGACCCTTCGGG-3). Unlabelled probes were used for competition experiments. The competition assay was performed by adding a 200-fold molar excess of the unlabeled probe. The extracts were incubated with DNA probes containing or lacking 8-oxoG and were resolved on a 4% polyacrylamide gel in 0.5 × TBE buffer. Supershift assays were carried out by incubating the extract with anti-NF-κB p65 antibody for 60 min prior to addition of double stranded DNA. To validate the effect of TH5487 on the binding of NF-κB to the DNA probe, different concentrations of TH5487 were incubated with the nuclear extracts before adding the probe.

### 2.12. Apoptosis, Necrosis and Viability Assays

Primary murine pancreatic acinar cells were plated in 96-well plates and treated with different concentrations of TH5487 for 1 h and then further co-incubated with 100 nM cerulein for 21 h. Cell survival (calcein assay) and apoptosis (caspase activity) were measured as described previously [22]. Necrotic cell death was assessed by the release of lactate dehydrogenase (LDH) as previously described and by Sytox green uptake. For the latter, cells were stained with Sytox green (0.5 µM) and DRAQ5 (2.5 μM) for 15 min. Images were analyzed with an Opera Phenix High-Content Analysis system (Perkin Elmer, Waltham, MA, USA) as previously reported [22].

### 2.13. Comet Assay

Alkaline comet assay was performed using Trevigen Flare slides as described previously [28]. Comets were analyzed with Open Comet software. Cells (*n* = 50) were randomly selected from all conditions for the statistical analysis.

### 2.14. Statistical Analysis

Results are presented as mean ± SD. Experimental data from different groups were compared by the unpaired *t*-test and one-way analysis of variance (ANOVA); and a value of *p* < 0.05 was considered statistically significant.

## 3. Results

**Figure 1:**
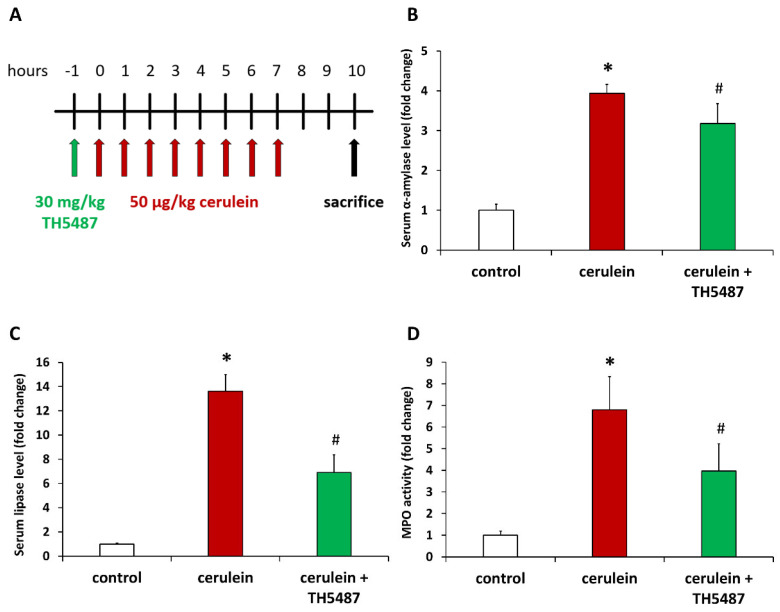
OGG1 inhibition reduces acinar cell injury and inflammatory cell recruitment in cerulein-induced acute pancreatitis. The treatment protocol is presented on panel. Mice were injected i.p. with TH5487 or vehicle 1 h before induction of AP with 8 repeated cerulein injections. Blood and pancreata were collected 10 h after the first cerulein injection when animals were sacrificed (**A**). TH5487 markedly reduced serum amylase (**B**) and lipase levels (**C**) and suppressed leukocyte infiltration in the pancreas as indicated by decreased MPO levels (**D**). Values represent mean ± SD, *n* = 6. * *p* < 0.05 vs. control group, # *p* < 0.05 vs. cerulein group.

In order to investigate the role of OGG1 in AP, we first set up an in vivo model of AP (Figure 1A). Repeated injections of the cholecystokinin analogue peptide cerulein induce pancreatic overstimulation and consequential AP-like symptoms. Indeed, cerulein caused elevated serum amylase and lipase levels reflecting acinar cell injury (Figure 1B,C). Treatment of mice with the OGG1 inhibitor TH5487 provided protection from pancreatic injury as indicated by reduced serum amylase and lipase activities (Figure 1B,C). Moreover, tissue levels of MPO, an indicator of monocytic/granulocytic infiltration of tissues, were also higher in the cerulein-treated group compared to controls (Figure 1D). TH5487 treatment significantly suppressed recruitment of white blood cells to the pancreas (Figure 1D).

**Figure 2:**
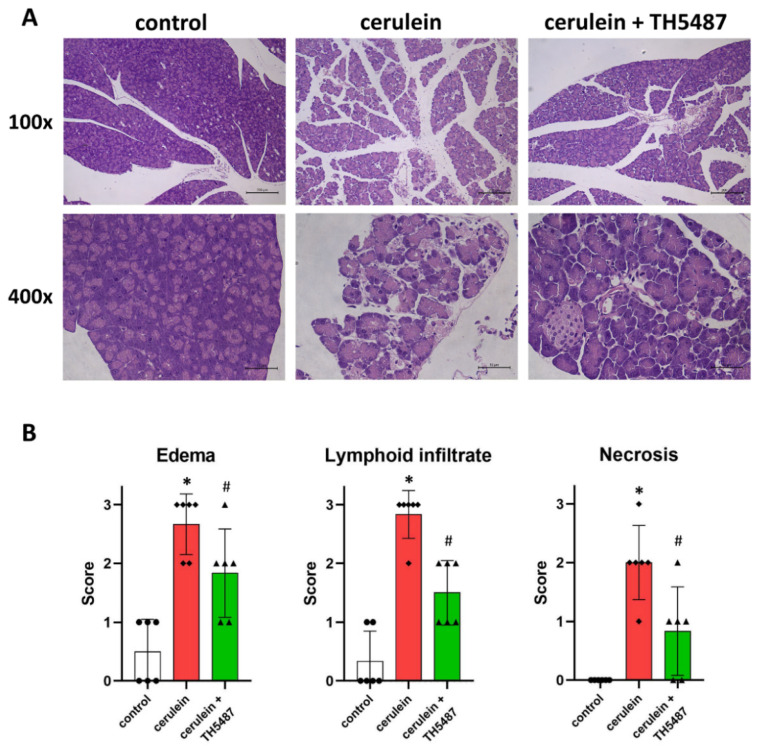
The OGG1 inhibitor TH5487 reduces inflammation and tissue injury of the pancreas. Representative images of H&E-stained pancreatic sections are presented (**A**). Tissue damage of the pancreas was observed histologically in the cerulein-treated group. TH5487 significantly reduced edema, inflammatory cell infiltration and tissue necrosis (cerulein + TH5487). The morphological changes in the pancreata were evaluated by an experienced pathologist (**B**). Magnifications were 100× and 400×. Scale bars represent 200 and 50 µm. Values are presented as mean ± SD, *n* = 6. * *p* < 0.05 vs. control group, # *p* < 0.05 vs. cerulein group.

Tissue samples obtained from pancreata underwent routine histology staining (H&E). Images obtained from the stained pancreas sections of cerulein-treated mice showed signs of edema, necrosis and leukocyte infiltration (Figure 2A). A trained pathologist evaluated the sections and scored them for these parameters (Figure 2B). These data revealed that the protective effects of TH5487 on edema formation, necrosis and leukocyte infiltration were all statistically significant compared to the cerulein-treated group (Figure 2B).

**Figure 3:**
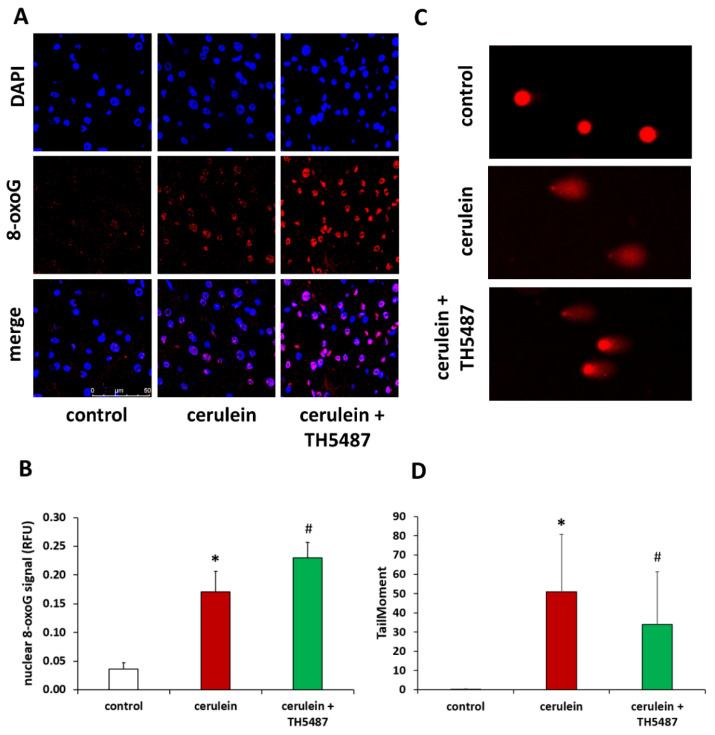
TH5478 increases 8-oxoG content but decreases DNA strand breakage. (**A**) Immunofluorescence staining of pancreas tissue sections for 8-oxoG (red) as the index of oxidative DNA damage. Nuclei were counterstained with DAPI (blue). Magnification was 400×. The scale bar is 50 µm. (**B**) The intensity of 8-oxoG increased significantly in the TH5487-treated group compared to the cerulein group. (**C**) The level of DNA strand breakage in primary acinar cells was detected by alkaline comet assay. Prior to the 100 nM cerulein treatment, cells were preincubated with 5 µM TH5487 for 1 h. (**D**) Cerulein treatment induced DNA breakage and OGG1 inhibition reduced the amount of DNA breakage. Values are presented as mean ± SD. * *p* < 0.05 vs. control group, # *p* < 0.05 vs. cerulein group*.

Cerulein-induced pancreatitis was characterized by a marked increase in DNA oxidation in the exocrine pancreas as indicated by immunofluorescent detection of the tissue’s 8-oxoG content (Figure 3A). Quantitative evaluation of the 8-oxoG staining confirmed that TH5487 caused a further increase in the accumulation of the oxidized nucleoside (Figure 3B). Similar changes in tissue 8-oxoG levels could also be confirmed by immunohistochemistry (Supplementary Figure S1). We hypothesized that the failure to excise oxidized guanine from DNA results in less DNA breaks. Indeed, comet assay experiments revealed a significant decrease in DNA breaks (Figure 3C,D). To prove that TH5487 treatment interferes with the repair of oxidative DNA lesions we induced DNA oxidation in AR42J pancreatic acinar cells by KBrO3 treatment and detected 8-oxoG by immunofluorescence (Supplementary Figure S2). We found that the level of 8-oxoG was elevated in the KBrO3-treated cells. Moreover, OGG1 inhibition by TH5487 resulted in a further increase in the cellular 8oxoG content (Supplementary Figure S2A,B).

**Figure 4:**
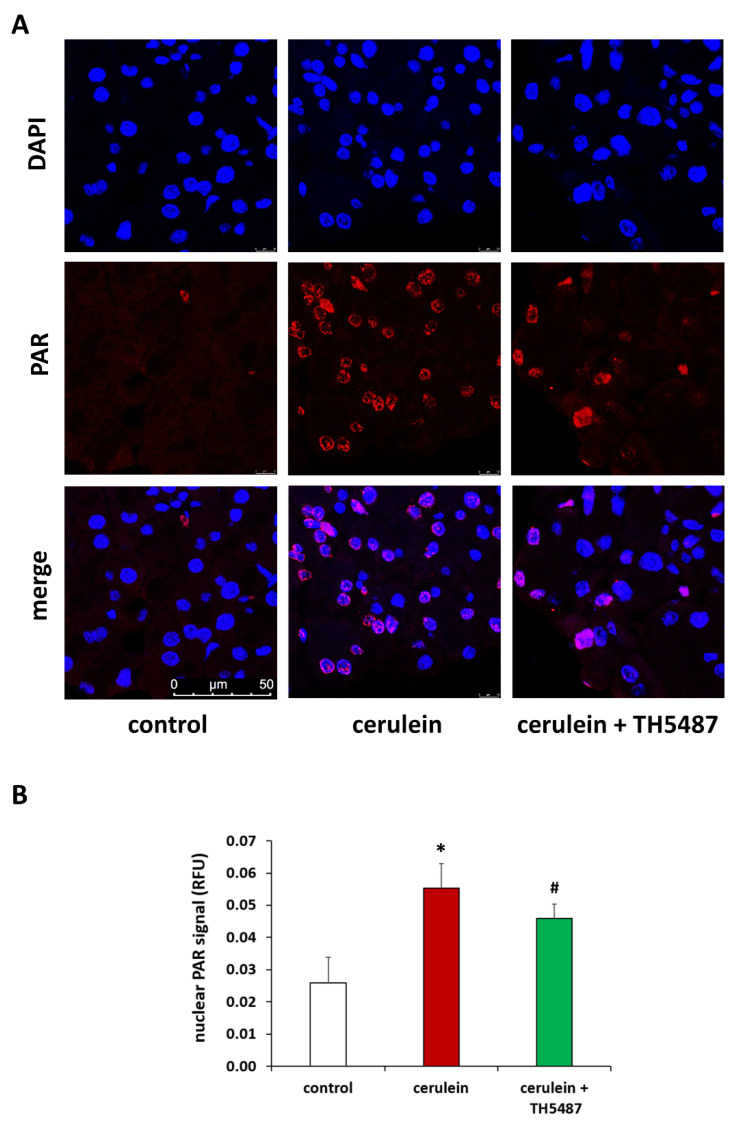
TH5478 reduces nuclear PARylation in AP. Representative images of pancreatic sections immunostained for poly(ADP-ribose) polymers (**A**). Nuclear PAR formation increased in cerulein-treated mice compared to the controls. PAR levels were significantly reduced after TH5487 treatment. Quantitation of PAR immunofluorescence signals (**B**). Values are presented as mean ± SD, *n* = 6. * *p* < 0.05 vs. control group, # *p* < 0.05 vs. cerulein group. Scale bars are 50 µm.

Based on the involvement of PARP1 in the repair of 8-oxoG lesions we also investigated how OGG1 inhibition affects poly(ADP-ribosyl)ation (PARylation) activity in our model. In line with previous findings [10,29], elevated PAR polymer levels could be detected in the pancreata of cerulein-treated mice by immunofluorescence (Figure 4). Furthermore, PARP1 expression levels were also elevated in the cerulein-treated group (Supplementary Figure S3). OGG1 inhibition decreased pancreatic PAR levels (Figure 4), whereas PARP1 levels were unaffected (Supplementary Figure S3).

**Figure 5:**
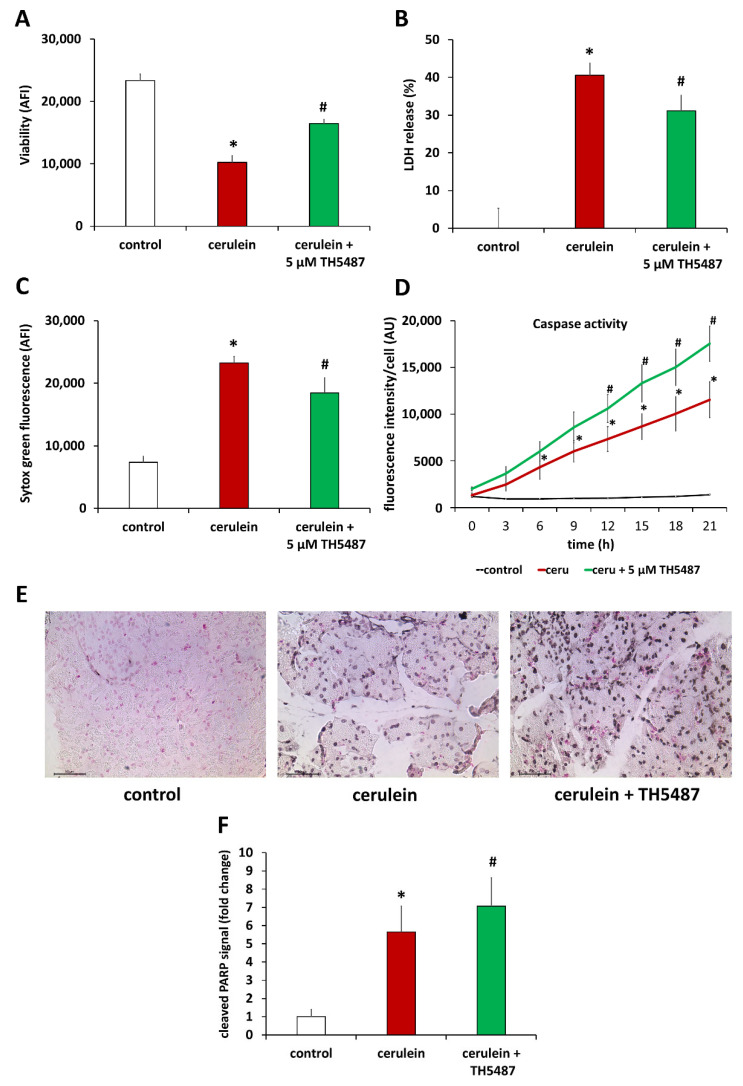
OGG1 inhibition increase cell viability by promoting apoptosis and reducing necrosis. (**A**) OGG1 inhibition increased viability in primary acinar cells and reduced necrosis as shown by decreased LHD level (**B**) and Sytox Green intensity (**C**). OGG1 inhibition increased apoptosis in primary acinar cells (**D**), which correlates to the results of immunohistochemical staining of pancreas sections for cleaved PARP (**E**). (**F**) Cleaved PARP formation increased in cerulein-treated mice compared to the controls and increased significantly further in TH5487 treatment group. Data are presented as mean ± SD. * *p* < 0.05 vs. control group, # *p* < 0.05 vs. cerulein group.

Based on the central role of PARP1 activation in oxidative stress-induced regulated necrotic cell death [30,31], we set out to investigate how lower PARylation activity translates to cell death in TH5487-treated animals. In line with our expectations, we found that cerulein decreased the viability of primary pancreatic acinar cells (Figure 5A). Cell death was of mixed type showing signs of both necrosis (LDH release and Sytox green uptake; Figure 5B,C) and apoptosis (caspase activity; Figure 5D). While OGG1 inhibition provided protection from loss of viability (Figure 5A) and from cell necrosis (Figure 5B,C), it resulted in elevated caspase activity (Figure 5D). The latter finding could also be confirmed in vivo as cleaved PARP-1 signal (a sign of caspase activity) was more intense in the pancreatic sections of TH5487-treated mice compared to the vehicle-treated group (Figure 5E,F).

**Figure 6:**
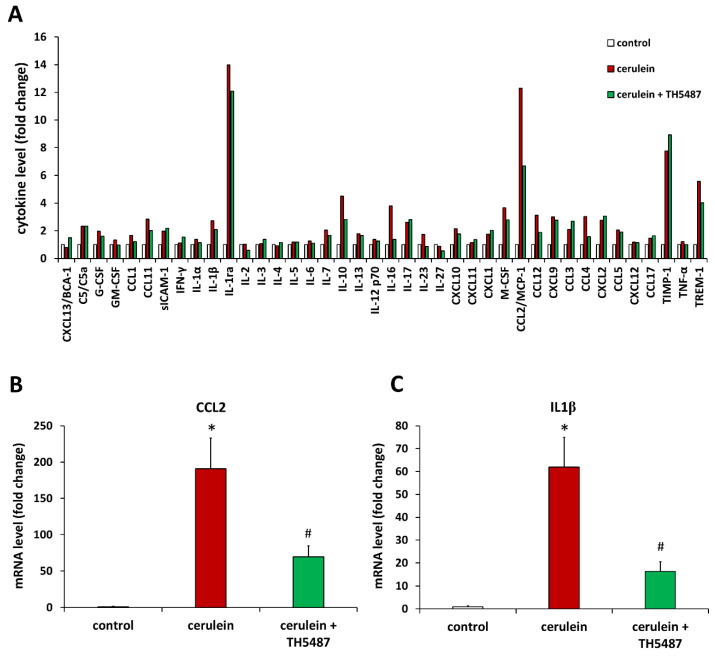

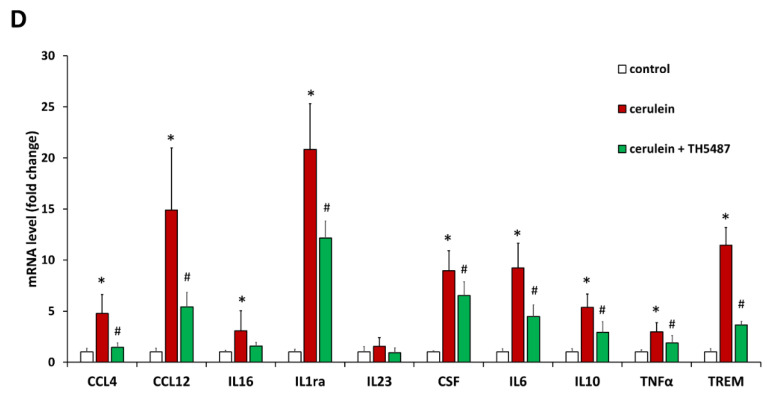
TH5487 prevents proinflammatory cytokine production in AP. Cytokine levels were measured with cytokine arrays from pancreas tissues obtained 10 h after the first cerulein injection. TH5487 markedly reduced the level of CCL2 (**A**). The relative expressions of cytokines and chemokines in pancreata were measured by qPCR. Cerulein caused a 190-fold increase in CCL2 mRNA level (**B**) and a 61-fold increase in IL1β mRNA level (**C**). The OGG1 inhibitor significantly suppressed both CCL2 and IL1 expressions (**B**,**C**). TH5487 also significantly reduced the expression of several other proinflammatory genes (**D**). qPCR data are presented as mean ± SD, *n* = 6. * *p* < 0.05 vs. control group, # *p* < 0.05 vs. cerulein group.

We also wanted to investigate how the tissue protective effect of the OGG1 inhibitor translates to inflammatory gene and protein expressions. Cytokine arrays were used to determine cytokine levels from pancreas tissues obtained 10 h after the first cerulein injection. Of the 40 cytokines and chemokines quantified with the array, 10 had at least 3-fold higher level in the cerulein-treated group compared to control (Figure 6A). Then we confirmed these data at the level of mRNAs and found that TH5487 treatment significantly reduced the levels of ten inflammatory marker mRNAs including those of IL1β, IL1ra, IL6, TNFα, and three chemokines of the CCL family (Figure 6B–D).

**Figure 7:**
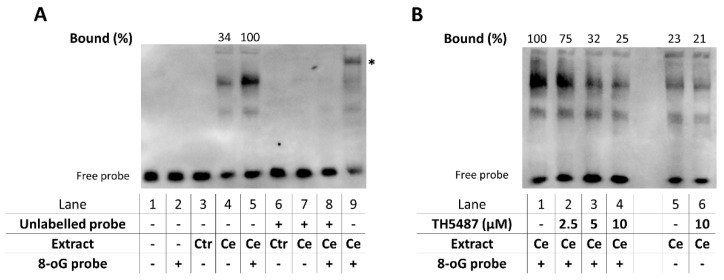
OGG1 inhibition reduces the amount of active NF-κB in AP and decreases NF-κB-binding to 8-oxoG-containing response elements. (**A**) Pancreatic nuclear extracts were prepared from mice treated with saline (Ctr) or cerulein (Ce). EMSA was performed by incubating the extracts with native or 8-oxoG modified NFκB-response element containing oligonucleotide probes. A mobility shift of the native probe confirmed that the Ce nuclear extracts contained active NF-κB (lane 4), whereas the Ctr extract caused no shift (lane 3). Nearly 3-fold increase was detected in NF-κB binding when a guanine by the response element in the probe was replaced with 8-oxoG (lane 5 vs. 4). The assay specificity was confirmed by competing off binding with an excess of unlabeled probe (lane 7–8) and by supershifting the probe with an anti-p65 antibody added to the Ce extract prior to the EMSA (lane 9, asterisk). (**B**) TH5487 when added to the Ce extract before the DNA, decreased NF-κB binding to 8-oxoG containing probes in a dose-dependent manner (lanes 1–4). TH5487 markedly decreased NF-κB DNA occupancy in the presence of 8-oxoG next to the response element (lane 4) but had no effect in the absence of 8-oxoG (lane 6). Data are representative of three experiments. Binding occupancies were calculated from band intensities using the Image J software. Relative band intensities were compared to the Ce extract incubated with the 8-oxoG probe, which was taken as 100% (lane 5 and lane 1 in panels A and B, respectively).

Since most inflammatory cytokines and chemokines that were upregulated in AP were inhibited by TH5487, we hypothesized that the OGG1 inhibitor likely targets a central pathway in inflammatory signaling. NF-κB is the master regulator of inflammatory gene expression in various models including AP and therefore we focused on NF-κB in our mechanistic experiments. Activation of NF-κB could be clearly demonstrated in EMSA experiments proving the presence of NF-κB in the nuclear extracts prepared from pancreatic tissues of cerulein-treated mice (Figure 7A). While the transcription factor bound to its response element, it showed an even more pronounced binding to the response element containing oxidized guanine bases (Figure 7A). We also tested the effect of the OGG1 inhibitor on this interaction and found that TH5487, when added to the EMSA assay, had a concentration dependent inhibitory effect on target site occupancy of NF-κB when tested with oxidized response elements (Figure 7B). While the binding of NF-κB was weaker to the 8-oxoG-free sequence than to the 8-oxoG containing ones, TH5487 had no effect on the binding to the 8-oxoG-free DNA fragments (Figure 7B).

## 4. Discussion

Oxidative stress defined as an imbalance between the production and elimination of ROS and RNS species is a common feature of all forms of inflammation. When the capacity of antioxidant mechanisms is exhausted, ROS and RNS can cause tissue damage by reacting with biomolecules such as proteins, lipids and DNA. These observations prompted numerous preclinical studies and clinical trials investigating the potential beneficial effects of antioxidant interventions ranging from the administration of natural and synthetic antioxidant compounds, inhibitors of ROS production pathways and PARP inhibitors.

Several studies have also implicated oxidative stress in the pathomechanism of acute pancreatitis. On the one hand, protein oxidation and lipid peroxidation have been reported in AP [32,33], while on the other hand antioxidant interventions often proved beneficial in animal models of AP [8,22,34,35,36,37,38,39]. Nonetheless, none of these interventions could be or have been efficiently translated to human AP.

Whereas ROS and RNS-induced direct and cumulative cell damage is an attractive concept to explain tissue injury in inflammation and ischemia-reperfusion injury of vital organs, recently novel signaling mechanisms have also been put forward to serve as underlying pathways of these ROS and RNS-related pathologies [40,41]. Moreover, some of these pathways also explain how ROS and RNS contribute to the inflammatory pathways. One of the most intriguing recent developments in redox pathobiochemistry has been the uncovering of how DNA oxidation is linked to inflammation. The pathway begins with the oxidation of guanine in DNA and RNA. Of all the nucleobases, guanine has the lowest redox potential, hence the most susceptible to oxidative damage [42,43]. Guanine oxidation in DNA results in the formation of 8-oxoG and these lesions are effectively repaired by OGG1-mediated base excision repair.

OGG1 has been linked to the inflammatory response (e.g., in in contact hypersensitivity (dermatitis) [18], endotoxin shock [18] and allergic lung inflammation [19]. The role of OGG1 in AP, however, has not yet been studied. The availability of the small molecule OGG1 inhibitor TH5487 permitted our investigations on the role of OGG1 in AP. Collectively, our findings suggest that OGG1 inhibition provides protection from both tissue injury and inflammation in AP. These statements are supported by our findings that TH5487 treatment lowered serum levels of tissue injury marker enzymes amylase and lipase, inhibited granulocyte migration to and edema formation in the pancreas (Figure 1 and Figure 2). One possible explanation for these findings is that interfering with 8-oxoG processing prevents activation of the DNA break sensor enzyme PARP1. Since intense PARP1 activation can mediate both cell death and inflammation, a suppressed PARP activation may have both tissue protective and anti-inflammatory consequences. Such roles of PARP1 have also been previously demonstrated in AP [44], however, the interrelationship between the OGG1 and PARP1 pathways in AP have not yet been investigated. Here, we also presented data indicating increased PAR synthesis in AP. Moreover, here we also showed that the OGG1 inhibitor treatment decreased pancreatic PAR content. It seems plausible to hypothesize that, when OGG1 is inhibited, 8-oxoG is not efficiently removed from the DNA and therefore the AP endonuclease will introduce less breaks into the affected DNA strands. Since DNA breaks are the primary triggers for PARP1 activation [30,45], less DNA termini would lead to lower PARP activation.

As for the reduced granulocyte migration of the TH5487-treated group (as reflected by tissue myeloperoxidase activity) it is important to note that chemokines such as CCL2 and CCL4 were among the genes most efficiently downregulated by TH5487 (Figure 5). Since CCL2 and CCL4 are major chemoattractants for granulocytes/monocytes, suppressed CCL2 and CCL4 expressions may have played a key role in the cell recruitment inhibitory effects of TH5487. Not only recruitment but activation of monocytes/granulocytes were also affected by the drug as reflected by reduced levels of the neutrophil/monocyte activating TREM1 receptor (Figure 5D). Moreover, the OGG1 inhibitor also significantly suppressed central inflammatory cytokines such as IL1β, TNFα and IL6 in the pancreas (Figure 5) and these effects likely played a key role in the general anti-inflammatory effects of TH5487.

Previous studies demonstrating an inflammation promoting role of OGG1 may provide clues for how this protein functions in AP. Animal models in which OGG1 has been shown to promote inflammation include contact hypersensitivity (skin inflammation; [18]), endotoxin shock (generalized, “whole body” inflammation) [18], asthma [46], Pseudomonas infection [47], hypoxia-reoxygenation-induced neuroinflammation [48] and TNF-induced lung inflammation [23]. Given the central role of NF-κB signaling in AP we considered this pathway as the most likely target of the OGG1 inhibitor. Activation of NF-κB could be confirmed by our EMSA experiments. Cerulein treatment caused nuclear translocation of NF-κB in the pancreas (Figure 6A). When oxidized guanin was present in the NF-κB response element, an increased binding of the NF-κB could be observed (Figure 6A). The OGG1 inhibitor TH5487 suppressed target site occupancy of NF-κB when tested with oxidized response elements (Figure 6B) but had no effect on the binding of NF-κB to the 8-oxoG-free DNA fragments (Figure 6B). Although our data are consistent with a model in which TH5487 interferes with DNA occupancy of NF-κB, additional mutually non-exclusive mechanisms can also be at play. For example, it has been reported that the 8-oxoG-OGG1 complex activates KRAS by acting as a GEF replacing KRAS-bound GDP with GTP [20]. This, in turn, activates the MAP kinase cascades fueling inflammation. Whether or not this mechanism is also active in our model goes beyond the scope of this work and requires further investigation.

In summary, OGG1 plays a role in the pathogenesis of AP. In AP, oxidative stress causes DNA oxidation resulting in the accumulation of 8-oxoG in the exocrine pancreas. OGG1 inhibition impairs the repair of 8-oxoG lesions resulting in less DNA nicks and consequently suppressed PARP1 activation. Interfering with PARP1 activation results in reduced acinar cell necrosis and a slight increase in apoptosis. In addition, 8-oxoG accumulation in the DNA may facilitate NF-κB binding to its consensus sequence in AP but when OGG1 is inhibited, target site occupancy of NF-κB is impaired. The protective effects of the OGG1 inhibitor in AP are likely due to interference with the PARP1 and NF-κB activation pathways. Our data support the potential therapeutic benefit of OGG1 inhibition in AP. Before taking any further steps towards the potential therapeutic use of OGG1 inhibition, we need to be sure that the procedure is safe. Unrepaired 8-oxoG is a mutagenic DNA lesion [49]. Knocking out the OGG1 gene has been shown to lead to increased mutation rates in the developing brain of gamma irradiated fetuses [50]. However, pharmacological inhibition of OGG1 may only lead to impaired but not fully blocked OGG1 function. Therefore, further experiments are clearly needed to investigate the long-term mutagenic potential of pharmacological OGG1 inhibition.

## Figures and Tables

**Table 1:** Primer sequences for qPCR.

Gene	Forward Primers (5→3)	Reverse Primers (5→3)
CCL2	CACTCACCTGCTGCTACTCA	GCTTGGTGACAAAAACTACAGC
CCL4	CCCACTTCCTGCTGTTTCTCT	GCCTCTTTTGGTCAGGAATACCA
CCL12	AAGCAGAAGATTCACGTCCG	ATCCAGTATGGTCCTGAAGATCAC
IL23	CCAGCGGGACATATGAATCTAC	TGTCCTTGAGTCCTTGTGGG
IL16	AACTCCTCTACTGACTCCGC	ACCCTGTTCTGTCCCTTTGA
IL10	GCTGTCATCGATTTCTCCCCT	GACACCTTGGTCTTGGAGCTTAT
CSF1	GCCCTTCTTCGACATGGCT	CCTTCAGGTGTCCATTCCCA
TREM1	TGCCACTTCATACACTGGGT	ATTGTGGAGAAGTCGAGGCA
IL1-ra	CATTGCTGGGTACTTACAAGGA	AGTGATGTTAACTTCCTCCAGC
IL6	TTCTTGGGACTGATGCTGGT	CCATTGCACAACTCTTTTCTCA
IL1β	TGCCACCTTTTGACAGTGATG	ATGTGCTGCTGCGAGATTTG
TNFα	GGATGAGAAGTTCCCAAATGGC	TGTCTTTGAGATCCATGCCG

## Data Availability

Data supporting reported results have been deposited in Zenodo (https://doi.org/10.5281/zenodo.6548205 (accessed on 15 March 2022)).

## Supplementary Materials

The following supporting information can be downloaded at: https://www.mdpi.com/article/10.3390/biomedicines10102543/s1, Figure S1: TH5487 treatment results in increased accumulation of nuclear 8-oxoG lesions. (A) Representative images of 8-oxoG staining in pancreas sections, (B) TH5487 results in accumulation of genomic 8-oxoG lesions in the pancreas. OGG1 inhibition increased further 8-oxoG level. Magnification was 400×. Scale bars are 50 µm. Values are presented as mean ± SD, *n* = 6. * *p* < 0.05 vs. control group, # *p* < 0.05 vs. cerulein group. Figure S2: TH5487 treatment leads to increased 8-oxoG content in KBrO_3_-treated AR42J cells. (A) Immunofluorescene staining of 8-oxoG in AR42J cell line. (B) The level of genomic 8-oxoG was significantly higher in potassium bromate-treated cells compared to the control. TH5487 treatment caused increased 8-oxoG content in KBrO_3_-treated cells indicating that TH5487 prevents 8-oxoG repair by OGG1. Data shown are representative of three experiments. Data are presented as mean ± SD, * *p* < 0.05, # *p* < 0.05. Scale bars are 75 µm. Figure S3: OGG1 inhibition has no effect on PARP1 expression. (A) Immunohistochemical staining of PARP in the AP model. (B) PARP expression levels increased by two fold in cerulein and cerulein + TH5487 treated groups. Values are presented as mean ± SD, *n* = 6. * *p* < 0.05 vs. control group.
