# Supplementary material for: OGG1 Inhibition Reduces Acinar Cell Injury in a Mouse Model of Acute Pancreatitis
Source: Biomedicines. 2022 Oct 12;10(10):2543. doi: 10.3390/biomedicines10102543 (PMC9599718; doi:10.3390/biomedicines10102543)
Supplement: Supplementary file 1 [file biomedicines-10-02543-s001.zip › biomedicines-1954805-supplementary.pdf]

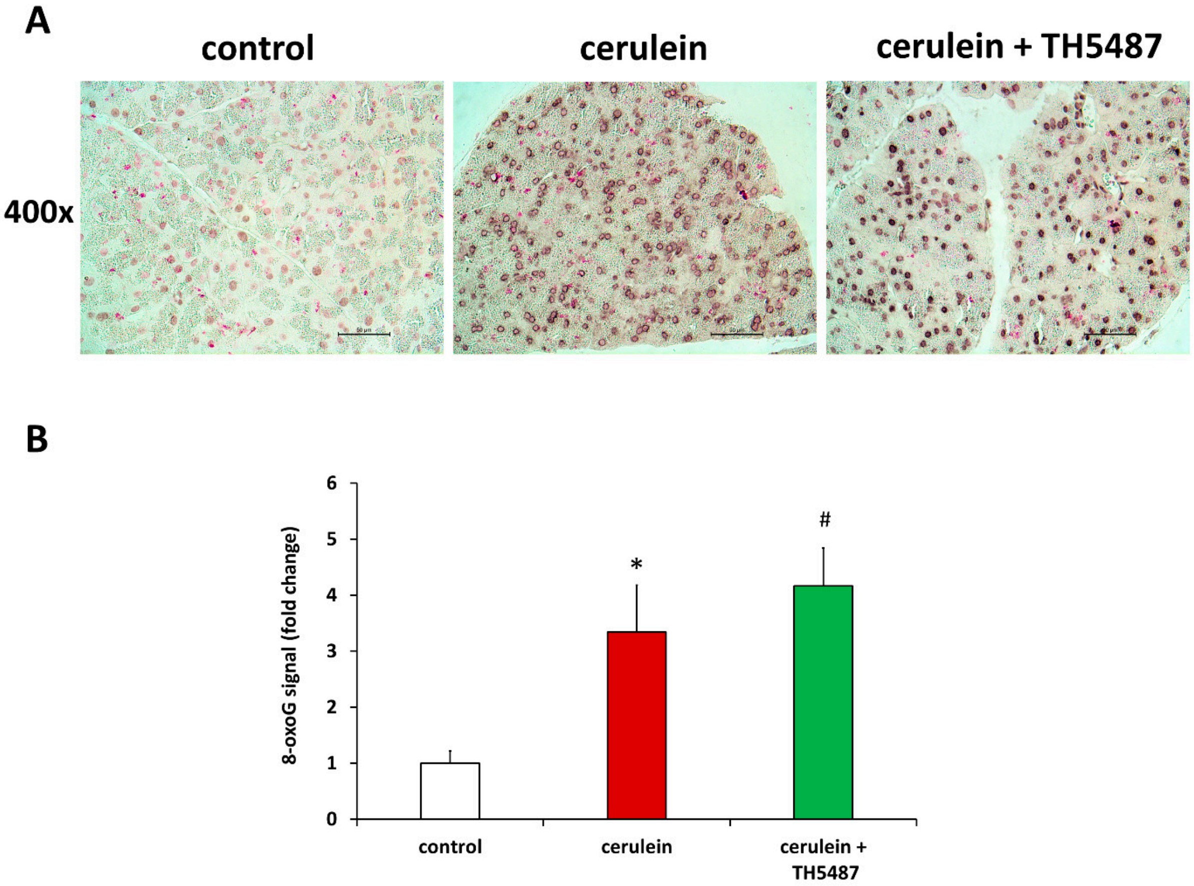

Supplementary Figure S1. (A) Representative images of 8-oxoG staining in panceas sections **(B)** TH5487 results in accumulation of genomic 8-oxoG lesions in the pancreas. OGG1 inhibition increased 8-oxoG level. Magnification was 400x. Scale bars are 50 µm. Values are presented as mean ± SD, n=6. *p<0.05 vs. control group, #p<0.05 vs. cerulein group

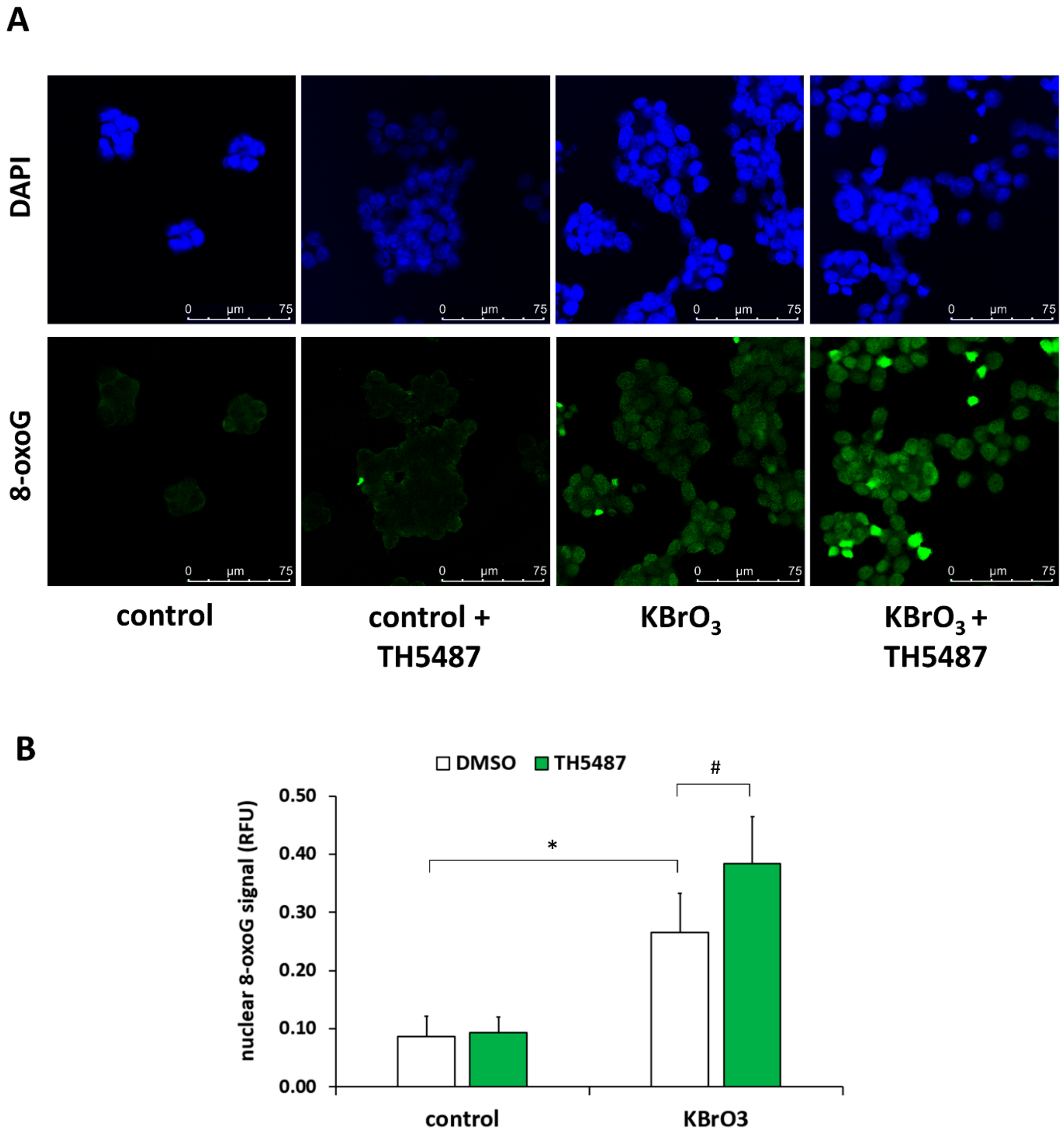

Supplementary Figure S2. (A) Immunfluorescent staining of 8-oxoG in AR42J cell line. Representative images of three experiments are shown. **(B)** The level of genomic 8-oxoG was significantly higher in potassium bromate-treated cells compared to the control. TH5487 treatment caused increased 8-oxoG formation in $KBrO_3$-treated cells indicating that TH5487 prevents 8-oxoG repair by OGG1. Data are presented as mean ± SD, *$P < 0.05$, #$P < 0.05$. Scale bars are 75 µm.

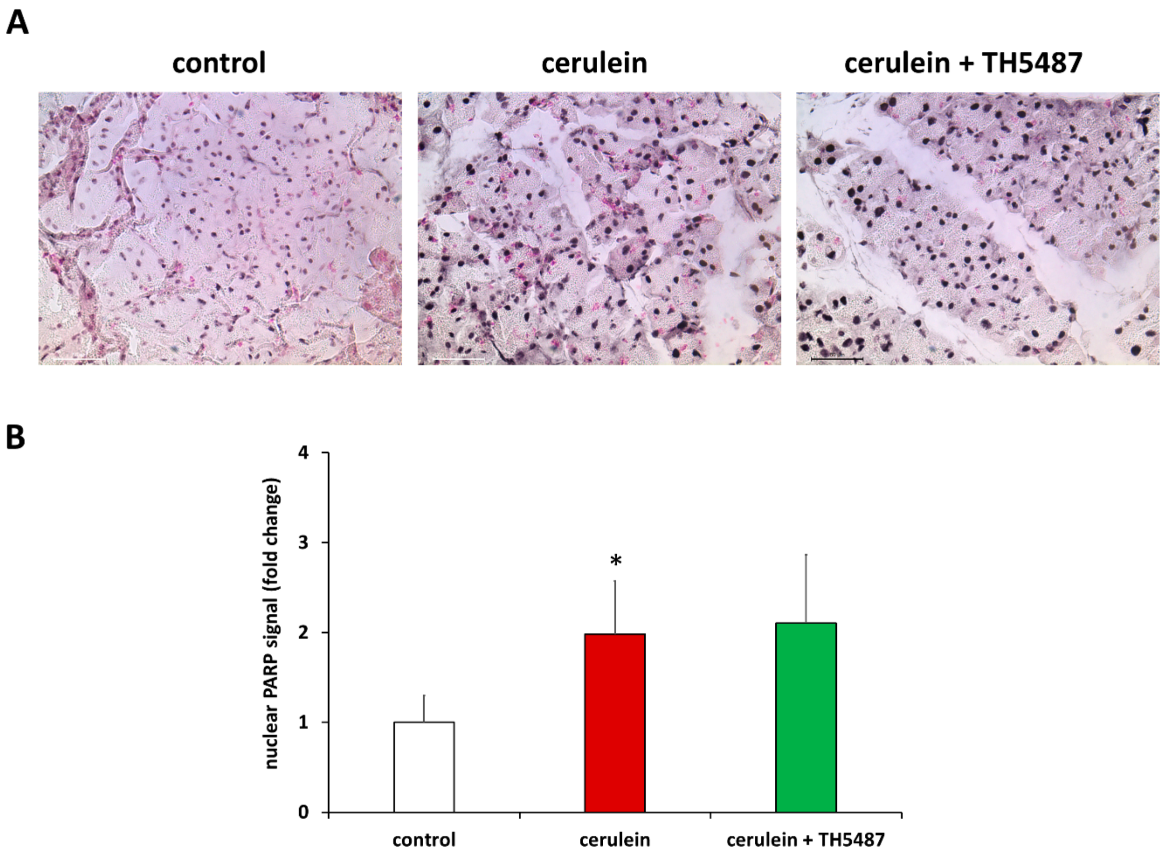

Supplementary Figure S3. (A) Immunhistochemical staining of PARP in AP modell. **(B)** The PARP amount increased by two fold in cerulein and cerulein + TH5487 treated group. Values are represented as mean ± SD, n = 6. *P < 0.05 vs. control group.
